# Diagnostic test performance of the Mentzer index in evaluating Saudi children with microcytosis

**DOI:** 10.3389/fmed.2024.1361805

**Published:** 2024-07-29

**Authors:** Amani M. AlQarni, Arwa Althumairi, Nourah K. Alkaltham, Samaa AlJishi, Amani Mohammed Hakami, Leena Mohamed Osman Ali Abdalla, Zahra Sayed Jalal Alawi, Abdullah H. Alreedy

**Affiliations:** ^1^Family and Community Medicine Department, King Fahd Hospital of the University, Imam Abdulrahman Bin Faisal University, Dammam, Saudi Arabia; ^2^Department of Health Information Management and Technology, College of Public Health, Imam Abdulrahman Bin Faisal University, Dammam, Saudi Arabia; ^3^King Fahad Hospital of University, Imam Abdulrahman Bin Faisal University, Alkhobar, Saudi Arabia; ^4^College of Medicine, Imam Abdulrahman Bin Faisal University, Dammam, Saudi Arabia; ^5^College of Medicine, University of Khartoum, Khartoum, Sudan, Khartoum, Sudan; ^6^Faculty of Medicine, Mansoura University, Mansoura, Egypt

**Keywords:** microcytosis, Mentzer index, diagnostic test performance, iron deficiency anemia, thalassemia

## Abstract

**Background:**

Anemia is a global public health concern, affecting both developing and industrialized countries at a rate of 39.8%. It is defined by low hemoglobin concentration, and anemia varies in severity based on age: <11 g/dL (6–59 months), <11.5 g/dL (5–11 years), and < 12 g/dL (12–14 years).

**Aim:**

This study evaluates the Mentzer index’s reliability in differentiating iron deficiency anemia from the thalassemia trait.

**Methods:**

A total of 434 children (≤16 years) with hemoglobin electrophoresis previously screened for microcytosis (MCV <80 FL) and an iron profile were included. Children with other hematological conditions were excluded.

**Results:**

Out of 434 children, 181 were diagnosed with thalassemia, and 345 had iron deficiency anemia. The Mentzer index showed 74% sensitivity and 63% specificity for the beta-thalassemia trait, with 61% sensitivity and 36% specificity for iron deficiency anemia. The beta-thalassemia trait group had the highest negative predictive value (98%), while iron deficiency anemia had the highest positive predictive value (79%).

**Conclusion:**

Our study, which is consistent with previous literature, suggests that the Mentzer index is not highly reliable in distinguishing iron deficiency anemia from the thalassemia trait among children in Saudi Arabia.

## Introduction

According to international organizations, 1.62 billion people worldwide suffer from anemia, making it one of the 10 most serious health problems. In 2019, the prevalence of anemia among children aged 6–59 months was 39.8% worldwide, with the highest prevalence of 60.2% in the African region. Although the prevalence of anemia has decreased from 48.0% in 2000 to 39.8% in 2019, it has remained stagnant since 2010. The World Health Organization (WHO) estimates the prevalence of anemia among children under 5 years of age to be 21.8% in Saudi Arabia (1).

Anemia is a public health problem in both developing and industrialized countries, but it is more prevalent in developing countries, especially among women and children, who are particularly vulnerable to its effects (2). Anemia is a multifactorial disease influenced by multiple etiologies, including nutritional deficiencies, genetics, and socioeconomic status, all of which are strongly associated with its prevalence. Furthermore, anemia in childhood has been linked to a greater risk of growth delay, poor cognitive and motor development, and a higher susceptibility to infections (2).

The WHO defines anemia in childhood based on hemoglobin (Hb) concentration levels, taking into consideration the age of the child. For children aged 6–59 months, a level of <11 g/dL is considered anemia, while for children aged 5–11 years and older children aged 12–14 years, the levels of <11.5 g/dL and < 12 g/dL, respectively, are considered anemia (3).

Anemia is typically caused by three main mechanisms: ineffective erythropoiesis, hemolysis, and blood loss. Many etiologies have been attributed to anemia, but the most common contributors are nutritional deficiencies, diseases, and genetic Hb disorders (4).

Iron deficiency is the most common cause of anemia, contributing to approximately 42% of cases in children under 5 years of age worldwide and 50% of cases in non-pregnant and pregnant women (4, 5). Conditions that require an increase in the number of red blood cells (RBCs) include growing infants or the development of a fetus during pregnancy. When there is insufficient dietary iron intake to meet iron needs, iron deficiency anemia (IDA) develops, as iron is an essential component of the Hb molecule, which affects the production of RBCs (2).

IDA is characterized by hypochromic, microcytic anemia, associated with lower levels of iron. Patients with IDA may present with symptoms such as pallor of the skin, dyspnea, fatigue, and headache (6).

IDA has several etiologies, including physiological, pathological, environmental, drug-related, and genetic causes. Physiological causes include rapid growth, pregnancy, and blood donation, while decreased absorption and chronic blood loss are considered pathological etiologies (6).

Various factors contribute to a child’s susceptibility to developing IDA, including premature birth or low birth weight, the use of non-iron-fortified formula or early introduction of cow’s milk in the first year of life, and exclusive breastfeeding without consistent consumption of iron-fortified food beyond 6 months of age (7).

The diagnosis of IDA can be achieved by measuring ferritin levels, as it is the first laboratory test to decrease in cases of iron deficiency (8). Ferritin is considered the most sensitive test for diagnosing IDA (7, 9). However, ferritin levels can be influenced by acute-phase reactants, leading to less accurate measurements under conditions of concurrent infection or inflammation (10).

Genetic Hb disorders, such as sickle cell trait and thalassemia, are among the three main contributors to anemia globally (4). Annually, more than 300,000 children are predicted to be born with sickle cell anemia or various forms of thalassemia, predominantly impacting regions in South and Southeast Asia (11).

In Gulf countries, including Saudi Arabia, the prevalence of thalassemia in children under the age of 5 years ranges from 0.25 to 33%, while in children aged 5 years and above, it is estimated to be 0.9% (12). In the western region of Saudi Arabia, thalassemia is one of the most common blood disorders, with a prevalence of 40%, including 4.69% in beta-thalassemia and 35.86% in alpha-thalassemia (α-thalassemia) (13).

Thalassemia is a genetic disorder characterized by decreased synthesis of alpha or beta chains of Hb. There are two types of thalassemia: α-thalassemia and beta-thalassemia, which can vary in severity. α-thalassemia occurs when one or more of the globin genes are mutated, and it is classified as silent carrier, α-thalassemia trait (α-TT), hemoglobin H (HbH) disease, and α-thalassemia major (Hb Bart). Beta-thalassemia, on the other hand, is classified as beta-thalassemia major (transfusion-dependent thalassemia or TDT), beta-thalassemia intermedia, and β-thalassemia trait (β-TT). Patients with TDT typically experience severe symptoms and require regular blood transfusions, while βTT patients can remain asymptomatic and lead a healthy life (14).

For the diagnosis of thalassemia, a physical examination and detailed medical and family history are required. Additionally, certain RBC indices can raise suspicion of thalassemia, specifically low mean corpuscular volume (MCV), low mean corpuscular Hb level (MCH), and normal red cell distribution width (RDW). However, to confirm the diagnosis, Hb electrophoresis and DNA analysis are necessary (15, 16).

RBC indices are also crucial in differentiating the causes of anemia, and numerous studies have explored their diagnostic significance. These include the Kerman index, Mentzer index, Shine and Lal index, and England and Fraser index. The Mentzer Index, first described in 1973 by William Mentzer, is calculated by dividing the MCV by the RBC (14).

Numerous studies have evaluated the reliability of the Mentzer Index in differentiating between beta-thalassemia and IDA. It has been shown to have a sensitivity of 98.7% and a specificity of 82.3% compared to other hematological indices. In other studies, it has also been considered a simple screening tool (17, 18).

### Purpose

This study aims to assess the sensitivity and specificity of the calculated Mentzer index to predict iron deficiency and thalassemia traits.

## Methods

### Study design

This study used a quantitative cross-sectional design based on a secondary dataset.

### Study population and study power

The retrospective data collection process involved accessing the hospital database to retrieve information on patients who underwent Hb electrophoresis and iron profile for microcytic RBCs as part of their complete blood count (CBC) from 2014 to 2021 at a single hospital. To ensure the appropriateness of the study sample, specific inclusion and exclusion criteria were applied. Patients who met the inclusion criteria were included in the study. Initially, there were 1,009 children in the pool, but ultimately, only a total of 434 patients were included.

### Inclusion criteria

The following are the inclusion criteria for the study:

Children aged 16 years and below who were previously investigated for microcytosis (MCV < 80 fl) using a hematology analyzer (Abbott Alinity HQ).

Patients who underwent Hb electrophoresis using capillary electrophoresis (Capillarys 2 Flex Piercing instrument).

Patients who had an iron profile with ferritin level measured in ng/ml (using Abbott Alinity I), serum iron level measured in ug/dl (using Abbott Alinity c), transferrin level measured in mg/dl (using Abbott Alinity c), iron-binding capacity measured in ug/dl (using Abbott Alinity c), and calculated transferrin percentage saturation.

Reference ranges for The iron profile were As follows: Serum iron (65–175 ug/dl for male subjects and 50–170 ug/dl for female subjects), transferrin level (186–388 mg/dL for girls and 180–391 mg/dL for boys In pediatric Age), and iron-binding capacity (250–400 ug/dl for all age and sex).

### Exclusion criteria

The following are the exclusion criteria for the study:

Patients with coexisting hematological conditions such as autoimmune hemolytic anemia, aplastic anemia, or lead intoxication.

Patients with the coexistence of β-TT and IDA in the same patient.

The study power was estimated using the G*Power application. With an effect size of 0.3, a probability of error of 0.05, and degrees of freedom of 5, the study power was calculated to be 0.99.

### Data analysis

Patient data including date of birth, sex, Hb level, hematocrits, MCV, MCH, mean corpuscular Hb concentration (MCHC), RBC value, RDW, white blood count (WBC), platelets, iron level, ferritin value, and Hb electrophoresis (Hb A2, Hb A) were collected from hospital databases, which was performed using capillary zone electrophoresis, and CBC was performed using Alinity HQ.

The Mentzer index was calculated by dividing MCV in Femtoliter (Fl) by RBC counts in millions per microliter (19, 20).

Mentzer index = mean corpuscular volume (in fL)/RBC count (in millions per microliter).


$$\mathrm{Mentzer}\;\mathrm{Index}=\frac{\mathrm{Meancorpuscular}\;\mathrm{volume}\;\left(\mathrm{in}\;\mathrm{fL}\;\right)}{RBC\;\mathrm{count}\;\left(\mathrm{in}\;\mathrm{millions}\;\mathrm{per}\;\mathrm{microliter}\text{.}\right)}$$

If the test results are>13, the patient will be classified as having IDA, while if the patient scores <13, then the patient will be classified as having a thalassemia trait (21). Laboratory standard tests for IDA and thalassemia are as follows:

- IDA occurs when ferritin level = < 4.63 ng/mL or transferrin saturation is <20%.
- Thalassemia occurs when serum ferritin is >12 ng/mL and the red blood cell count is high.
- Alpha (à)*thalassemia trait: (* suspected cases of α-thalassemia, as confirmation can only be performed by molecular genetic study):

1. HbA2 < 4%
2. HbF < 1%

- β-TT:

1. HbA2 = > 4%
2. HbF 0.1–5%

All data were analyzed using a descriptive analysis; frequency and percentage for categorical data (sex and age group) and median and interquartile range (IQR) for continuous data (as data were not normally distributed). For the bivariate analysis, the chi-square test was used to compare the significant difference between the Mentzer index and the laboratory test.

## Results

**Figure 1:**
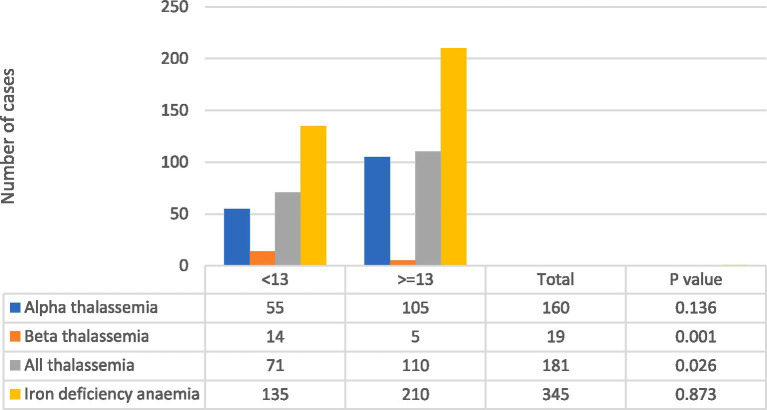
Percentage of cases by anemia groups based on the Mentzer index.

In this study, a total of 434 cases were included, as shown in Table 1. Among these cases, 345 were confirmed to have iron deficiency, while 181 were suspected to have thalassemia. Out of the suspected thalassemia cases, only 19 were confirmed to have the β-TT, as indicated in Figure 1.

**Table 1:** Distribution of patient demographic characteristics according to Mentzer classification, *n* = 434.

Patient characteristics	Calculated Mentzer index				Total *n* = 434	
	<13 *n* = 169		> = 13 *n* = 265			
	*N*	%	*N*	%	*N*	%
**Sex (*n*,%)**						
Male	42	25%	89	34%	131	30%
Female	127	75%	176	66%	303	70%
**Age *n* (%)**						
>2 years–6 years	65	38%	88	33%	153	35%
7 years–12 years	45	27%	74	28%	119	27%
13 years–16 years	59	35%	103	39%	162	37%
**Transferrin (median, IQR) (min-max)**	0.34 (0.11–7.26)	(0–48.0)	0.26 (0.1–8.4)	(0–92.4)	0.3 (0.1–7.6)	(0–92.5)
**Hemoglobin Electrophoresis A2 (median, IQR) (min-max)**	2.4 (2.0–2.7)	(0–6.9)	2.5 (2.1–2.7)	(0–5.9)	2.4 (2.1–2.7)	(0–6.9)
**Ferritin value (median, IQR) (min-max)**	19.0 (4.5–37.2)	(0–200)	22.0 (8.3–106.4)	(0–10930.0)	21.0 (6.7–56.0)	(0–10930.6)

In terms of sex distribution, the majority of cases were female subjects, accounting for 70% of the study population. When considering the age groups, the highest proportion of cases (37%) fell within the 13–16-year-old group. Among the groups classified with a Mentzer index <13, the proportion of female subjects was higher at 75% compared to 66% in the > = 13 group. On the other hand, male subjects were more prevalent in the > = 13 group at 34% compared to 25% in the <13 group.

Regarding the age distribution within the Mentzer index categories, the majority of children classified as Mentzer index <13 belonged to the 2–6-year-old age group, accounting for 38% of these cases. For the Mentzer index >13 group, the majority fell within the 13–16-year-old age group, representing 39% of the cases, as shown in Table 1.

Using the Mentzer index as a diagnostic tool, it was found that 71 out of the 181 suspected thalassemia cases had a score < 13, while 135 out of the 345 cases were classified as having a score > =13, indicating IDA, as depicted in Figure 1. Notably, the highest number of confirmed beta-thalassemia cases were classified as <13 using the Mentzer index, with only 5 cases falling into the > = 13 group.

In terms of the performance of the Mentzer index, it showed the highest sensitivity and specificity scores for the β-TT, with a sensitivity of 74% and a specificity of 63%, as presented in Table 2. Iron deficiency showed a sensitivity of 61% and a specificity of 36%. Similarly, the negative predictive value (NPV) was the highest for the beta-thalassemia group at 98%, while the positive predictive value (PPV) was the highest for IDA at 79%.

**Table 2:** Sensitivity and specificity of the Mentzer index for each disease classification.

Disease type	Analysis measure	Percentage
Beta-thalassemia	Sensitivity	74%
	Specificity	63%
	PPV	8%
	NPV	98%
Alpha-thalassemia	Sensitivity	34%
	Specificity	58%
	PPV	33%
	NPV	60%
All thalassemia	Sensitivity	39%
	Specificity	61%
	PPV	42%
	NPV	58%
Iron deficiency anemia	Sensitivity	61%
	Specificity	38%
	PPV	79%
	NPV	20%

## Discussion

The purpose of this research is to study the Mentzer Index and its reliability as a simple, inexpensive diagnostic tool in children aged 16 and younger in Saudi Arabia for differentiating between IDA and thalassemia traits. Although IDA and thalassemia are both types of conditions that can cause microcytic anemia, each type has a different cause, prognosis, and treatment (22).

The Mentzer index was first described by Mentzer in 1973 and is calculated by dividing the MCV by the RBCs (23). A Mentzer index value of lower than 13 shows that the patient has thalassemia, while an index greater than 13 suggests that the patient has IDA (24).

Based on the Mentzer index, our data indicate that 181 of the total cases were classified as thalassemia, while 345 were classified as IDA. The sensitivity was higher in the beta group (74%), and the specificity was 61% for all thalassemia groups.

Our results suggest that the Mentzer index has high sensitivity (74%) and higher specificity (63%) for identifying patients with beta-thalassemia. Contrarily, it showed low sensitivity for α-thalassemia, where the sensitivity is 34% and the specificity is 58%. Overall, the Mentzer index results suggest low sensitivity (39%) and high specificity (61%) for all thalassemia patients.

Regarding IDA, analysis results show that the sensitivity and specificity of the Mentzer index results were 61 and 38%, respectively. Hence, these findings indicate that the Mentzer index is more reliable in diagnosing IDA with a PPV of 79%, excluding βTT with an NPV of 98%, and suspected αTT with an NPV of 60%.

Our results are inconsistent with the findings reported by Ahmed et al. where the sensitivity and specificity of the Mentzer index were 95.24 and 93.10%, respectively, compared to 61% sensitivity and 38% specificity for IDA, and 74 and 63% of sensitivity and specificity, respectively, for βTT in our results (25). Similarly, our results differed from those of Vehapoglu et al., who retrospectively analyzed 290 children with microcytic anemia using the Mentzer index and showed good sensitivity (82.3, 98.7%) and specificity (98.7, 82.3%) for both the IDA and βTT groups. Furthermore, a study conducted by Ahmed et al. enrolled patients with microcytic hypochromic anemia aged from 6 months to 16 years and found that the Mentzer Index is a tool with one of the most reliable discriminator indices for differentiating β-TT and IDA in Sohag County (22). On the contrary, Düzenli Kar et al. reported that the Mentzer index was not determined to be one of the most reliable indices in their study (26). Similarly, Sirdah et al. reported that the Mentzer index was not the best among the evaluated indices in their study (27).

Similarly, a study published by Sherali et al. collected data from Liaquat National Hospital, Karachi, from 1st January to 30th June 2022 for children aged 1 to 5 years. They found that the Mentzer index has high sensitivity, specificity, and diagnostic accuracy, with 80.7% sensitivity and 77.7% specificity, and the PPV was 56.8%, while the NPV was 91.6% (28). Besides, Sri L. S. Alam et al. recommended the use of the Mentzer index in children aged 6–12 years with hypochromic-microcytic anemia (29). Additionally, a study was conducted to differentiate between βTT and iron deficiency anemia in randomly selected Croatian children aged 6 months–18 years, who were diagnosed with microcytic hypochromic anemia, found that the Mentzer index correctly diagnosed children with βTT, but was unsuccessful in distinguishing children with IDA (sensitivity 88%, specificity 48%), and thus, categorized the Mentzer index as low validity as it has a low proportion of correctly identified IDA (30).

In another study, where 200 children were enrolled, the sensitivity and specificity of the Mentzer index for the detection of βTT were 100 and 69.4%, respectively, while the PPV of the Mentzer index in diagnosing βTT was 36.6% and the NPV was 100%. Thus, the Mentzer index was found to be a valuable tool in differentiating IDA from TT (31). Finally, in a study that included 63 children with microcytic anemia aged between 2 and 16 years in 2002, Demir et al. reported somewhat similar results to our findings in the present study (see Tables 3, 4) (31).

**Table 3:** The Mentzer index results compared to other studies IDA.

Study	Sensitivity	Specificity	PPV	NPV
Current study	61%	38%	79%	20%
Ahmed et al.	95.24%	93.10%	90.9%	96.4%
Vehapoglu et al.	82.3%	98.7%	98.2%	86.3%
Ahmed et al.	100.00%	58.76%	23.95%	100%
Düzenli Kar et al. (IDA/α-TT)	64.52%	92%	82.6%	79.3%
Sirdah et al. (IDA/ 𝛽-TT)	82.86%	83.55%	–	–
Sherali et al.	80.7%	77.7%	56.8%	91.6%
Sri L. S. Alam et al.	93%	84%	93%	84%
Turudic et al.	88%	48%	-	-
Aydogan et al.	67.53%	97.36%	99.07%	44.04
Demir et al.	62%	86%	76%	76%

**Table 4:** The Mentzer index results compared to other studies 𝛽-TT.

Study	Sensitivity	Specificity	PPV	NPV
Current study	74%	63%	8%	98%
Ahmed et al.	95.24%	93.10%	90.9%	96.4%
Vehapoglu et al.	98.7%	82.3%	86.3%	98.2%
Ahmed et al.	100.00%	58.76%	23.95%	100%
Düzenli Kar et al. (IDA/α-TT)	80%	92%	90.9%	82.1%
Sirdah et al. (IDA/ 𝛽-TT)	82.86%	83.55%	–	–
Sherali et al.	80.7%	77.7%	56.8%	91.6%
Sri L. S. Alam et al.	93%	84%	93%	84%
Turudic et al.	88%	48%	–	–
Aydogan et al.	100%	69.48%	36.59%	100%
Demir, et al	86%	62%	76%	76%

According to Sri L. S. Alam et al., a screening tool is considered good if the sensitivity percentage is ≥80% , regardless of the low specificity percentage (29). According to Demir et al., studies performed to examine the index’s reliability in discriminating between IDA and thalassemia in children are limited, and the results of these studies are contradictory (32). In addition, no index with 100% sensitivity and specificity results has been reached for discrimination purposes to date. Furthermore, a low number of studies in the literature were concerned with α-thalassemia; therefore, it was not possible to compare our results with regard to the α-TT.

### Variability in sensitivity and specificity

This study observed a higher sensitivity (74%) for the Mentzer index in identifying β-thalassemia compared to IDA (61%). The specificity for β-thalassemia was also higher (63%) compared to IDA (38%). These findings were consistent with some studies [e.g., (1, 22)]. However, they differ from others, where high values for both IDA and β-thalassemia were reported [e.g., (2, 3)].

*Several factors might contribute to these* var*iations:*

- Population differences: the prevalence of specific thalassemia traits (α vs. β) and coexisting conditions such as iron deficiency can vary geographically and ethnically (4). This can affect the index’s performance.
- Age groups studied: studies focusing on specific age groups (e.g., children vs. adults) might show different results due to physiological variations in RBC parameters across lifespans (5).
- Iron deficiency severity: the severity of iron deficiency can influence RBC indices, potentially impacting the Mentzer Index’s discriminatory power (6).
- Analytical techniques: Variations in laboratory techniques used for CBC measurements might introduce slight discrepancies in the derived parameters (7).

Our findings align with studies highlighting the limitations of the Mentzer index as a standalone diagnostic tool (7, 8). While it shows promise for β-thalassemia detection (high sensitivity), its effectiveness for IDA diagnosis is lower (moderate sensitivity and low specificity). Additionally, the index performs poorly for α-thalassemia (low sensitivity).

Future research could explore the following areas:

- Combined Indices: Investigating the combined use of the Mentzer Index with other red blood cell parameters or iron studies to enhance diagnostic accuracy.
- Population-Specific Cutoff Values: Establishing optimal sensitivity and specificity thresholds for the Mentzer Index within specific populations, considering the local prevalence of thalassemia traits and iron deficiency anemia.
- Cost-Effectiveness Analysis: Comparing the cost-effectiveness of the Mentzer Index as a screening tool against other established methods for diagnosing iron deficiency anemia and thalassemia traits.

## Conclusion

In summary, it is important to evaluate the reliability of diagnostic tests to confirm the presence of disease in patients, rule out diseases in healthy individuals, and set a treatment plan. The analysis of our results shows lower sensitivity and specificity to distinguish between TT and IDA than those in the literature. The present study indicates that the Mentzer index is not a highly reliable discriminator index for differentiating between TT and IDA in children in the Kingdom of Saudi Arabia. One limitation of our study was the small sample size of patients. Consequently, more accurate research is needed to evaluate the best diagnostic index that can accurately differentiate between TT and IDA in children in the Kingdom of Saudi Arabia.

## Data availability statement

The datasets presented in this article are not readily available because sharing the raw data could potentially identify individual participants, which might put them at risk or violate their privacy. We prioritize upholding our ethical obligations and protecting the anonymity of our study participants. Requests to access the datasets should be directed to AMA, Amqurni@iau.edu.sa.

## Ethics statement

The studies involving humans were approved by Imam Abdulrahman bin Faisal University IRB-2021-01-334. The studies were conducted in accordance with the local legislation and institutional requirements. Written informed consent for participation was not required from the participants or the participants’ legal guardians/next of kin in accordance with the national legislation and institutional requirements.

## Author contributions

ArA: Conceptualization, Methodology, Validation, Writing – original draft, Writing – review & editing, Data curation, Formal analysis, Visualization. AMA: Conceptualization, Methodology, Validation, Writing – original draft, Writing – review & editing, Investigation. NA: Writing – original draft, Writing – review & editing. SA: Writing – original draft, Writing – review & editing. AH: Writing – original draft, Writing – review & editing. LA: Data curation, Validation, Writing – original draft, Writing – review & editing. ZA: Writing – original draft, Writing – review & editing. AHA: Writing – original draft, Writing – review & editing.
